# Supplementary material for: Biased expectations about future choice options predict sequential economic decisions
Source: Commun Psychol. 2024 Dec 18;2:119. doi: 10.1038/s44271-024-00172-8 (PMC11655870; doi:10.1038/s44271-024-00172-8)
Supplement: Supplementary file 2 — Supplemental material [file 44271_2024_172_MOESM2_ESM.pdf]

Supplementary Methods

Attention check

Attention checks were added to phase one (i.e., the ratings phase) of Pilot full and the Study 1 full and Study 1 ratings conditions, to compensate for the unsupervised nature of online data collection. Every attention check showed a cross, a ‘next ’button, and the text "press ‘next ’when the cross disappears". The cross disappeared at a random time interval between one and five seconds. The ‘next ’button was active the whole time. If participants were paying attention, they would not press the ‘next ’button as soon as it appeared but would instead read the text and respond only after the cross had disappeared. Thus, if participants’ response time exceeded the cross-display time, they passed the attention check. Nevertheless, we found high correlations between phase 1 and phase 2 ratings (i.e., > .8) across our studies (see average correlations between phase1 ratings reported in Methods to pilot studies and Study 1) and so we elected to not remove participants based on attention check data in Pilot full, Study 1 full and Study 1 ratings and we discontinued the use of attention check trials in Studies 2 and 3.

Detail on backwards induction

The action value associated with continued sampling (and, therefore, effectively the decision threshold for each sequence position) was computed based on a backwards induction algorithm. Here, we give an intuitive description of this algorithm, using the simplest incentivisation scheme, where the reward value of each option equals its option value and there is zero cost to sample. Backwards induction begins by considering the final option. When the agent encounters the final option, the decision threshold T_1 must be zero, as the paradigm forces choice of this option and there are no future option values to consider. Before that, when the agent encounters the second to last option, the computation of the threshold T_2 for this option remains relatively simple: the agent must compare the value of the second to last option against the *expected value* of the final option (i.e., the mean of generating distribution of option values). For the third to last option, its decision threshold T_3 depends on the expected value of continuing to the second to last option, with the opportunity to also continue to the final option. Computation of T_3 therefore involves integrating the density function of the generating distribution of option values to compute the expected value of options above the decision threshold T_2 and then adding the expected reward for the final position. This process can continue backwards to obtain the expected value of getting any option in the future that is better than the current one, wherever in the sequence the current one is.

Supplementary Results

Parameter recovery

To ascertain the ability of our models to derive the correct parameters from individual participant data, we performed parameter recovery analyses, in which we simulated model choices (take option or sample again) in response to randomly generated option values. We simulated models with 12 possible levels of each parameter, within which there were 25 simulated participants with five sequences per participant and 12 options per sequence. To parallel the structure of our empirical paradigms, we created a generating distribution (separately for each simulated participant) of 90 option values, randomly produced from a Gaussian distribution with mean 40 and standard deviation of 20 and within the range of 1 to 100 (recall that we normalised all our prices to this same range when fitting models to human participants). Then, we populated the sequences of input option values for the optimal stopping task from this participant-specific generating distribution. We configured our models with ranges of the key theoretical parameters (Supplementary Figure 4, x axis) that produced sampling rates between roughly two and ten samples to decision (Supplementary Figure 5). The aforementioned randomly generated option values were then presented to every configured model to extract simulated sampling rates associated with each configured parameter value. We then fitted the models to these simulated take option / sample again decisions in the same way as we fitted human participants to obtain parameter estimates of the configured parameters. For the three models we cover in the main text, Cost to Sample, Cut Off and Biased Prior models, configured and estimated parameters tended to correlate (Supplementary Figure 4 and lower panel of Supplementary Figure 5). Also, the sampling rates simulated using configured parameters highly correlated with sampling rates simulated using the estimated parameters (middle panel of Supplementary Figure 5 and Supplementary Figure 6).

We formulated two more theoretical models, but these did not perform so well during parameter recovery and so were excluded from model fitting and comparison. In the *Biased Values*

model, we considered the possibility that, although participants may use the optimal solution to solve the task, they might instead be biased to misperceive the magnitudes of the option values that are input into this optimal solution. This might especially be the case if participants perceive only the very most valued options as worthy of consideration at all, as might be the case in “high threshold” models of optimal stopping in mate choice[1]. We incorporated a threshold into the Biased Values model: Option values above this option value threshold are transformed to 100 (the maximal option value) and option values below this threshold are transformed to 1 (the minimal option value). The transformed option values, once rendered attractive only when above threshold, are then submitted to the Ideal Observer. We picked the centre of the input value range (i.e., 50) as the starting value for the threshold parameter when fitting to participants’ choices and bounded the parameter fitting to be within the option value range 1 to 100.

The *Biased Rewards model* is based on similar logic as the Biased Values model. However, instead of assuming participants place a threshold on the option values being input to the model, we instead assumed such a threshold on the reward function *R* (See Equation 3 in Methods). Recall that this function assigns reward payoffs the relative ranks of choices. As with the Biased Values model, we adopted a threshold, which was the free parameter, above which choices received a maximal reward of 100 and below which choices received a reward of 1. As in the Biased Values model, the starting value of this threshold parameter was initialised at 50 and fitting was bounded between 1 and 100.

Model Recovery

To confirm the validity of our model fitting procedures, we employed out-of-sample testing of model performance. We simulated 1000 participants’ data using the Cost to Sample, Cut Off and Biased Prior models. Each simulated participants’ models processed five sequences of options created in the same way as for the parameter recovery. Parameters (the first theoretical parameter)

and beta (the Softmax inverse temperature) were sampled from uniform distributions using the same ranges we used to simulate model behaviour in the parameter recovery. Then, the same three models were fit to all simulated participants' models. The results[2] (Supplementary Figure 7) show successful overall recovery, with the simulated models largely better (smaller) BIC than other fitted models, despite a small degree of confusability between Cost to Sample and Biased Prior models.

Supplementary Discussion

Divergent model predictions

As mentioned in the Discussion in the main text, all the models we tested excelled at predicting participants' mean sampling rates. Our supplementary analyses of the large sample in Study 2 further shows that individual differences in participant sampling rates were highly correlated with sampling rates predicted by all three models (Supplementary Figure 12). And, participants' sequence specific thresholds were approximated by all three models (Supplementary Figure 13). The models did, however, diverge in their predictions of participants' mean rank of chosen options (Supplementary Figures 8-11, 14). Here, the Cut Off heuristic was unable to obtain similar levels as participants, while Biased Prior and Cost to Sample models could. Given the high predictivity of all our models, it can be difficult to discern exactly what choices the Biased Prior is superior at predicting, compared to other models. Our recommendation is that these models be compared on paradigms specifically designed to test this hypothesis. For example, manipulations of participants' expectations about upcoming option values (i.e., their prior) should produce the types of systematically different decisions that would be predictable from a Biased Prior model.

Supplementary References

1. Valone, T.J., Nordell, S.E., Giraldeau, L-A. & Templeton, J.J. (1996). The empirical question of thresholds and mechanisms of mate choice. *Evolutionary Ecology, 10,* 447–455. https://doi.org/10.1007/BF01237729

2. Wilson, R. & Collins, A.E. (2019). Ten simple rules for the computational modeling of behavioral data. *eLife, 8,* e49547. https://doi.org/10.7554/eLife.49547

3. Baumann, C., Singmann, H., Gershman, S. J., & von Helversen, B. (2020). A linear threshold model for optimal stopping behavior. *Proceedings of the National Academy of Sciences 117,* 12750–12755. https://doi.org/10.1073/pnas.2002312117

Supplementary Tables

Supplementary Table 1

Details of pairwise tests comparing human participant and Ideal Observer sampling

Ideal Observer	n	Bayes Factor 10 (BF10)			t test	Cohen's d	Confidence interval		Lilliefors
		r = .5	r = .71	r = 1					
Pilot baseline									
Objective values	47	2.98E18	4.04E18	5.30E18	t(46) = -17.24, p < .001	-2.52	-3.89	-3.08	D = .12, p = .10
Pilot full									
Objective values	50	.41	.31	.23	t(49) = 1.23, p = .45	.17	-0.26	0.84	D = .10, p = .55
Subjective values	50	12.86	11.37	9.31	t(48) = 3.15, p = .01	.45	0.18	1.13	D = .09, p = .86
Study 1 baseline									
Objective values	50	7.00E15	9.31E15	1.18E16	t(49) = -14.19, p < .001	-2.01	-3.33	-2.5	D = .06, p = .50
Study 1 squares									
Objective values	50	1.9E13	2.49E13	3.04E13	t(49) = -12.10, p < .001	-1.71	-3.29	-2.35	D = .07, p = .50
Study 1 timing									
Objective values	50	3.7E13	4.86E13	5.97E13	t(49) = -12.33, p < .001	-1.74	-3.32	-2.39	D = .09, p = .36
Study 1 payoff									
Objective values	51	6.41E15	8.51E15	1.07E16	t(50) = -14.01, p < .001	-1.96	-3.98	-2.99	D = .08, p = .50
Study 1 ratings									
Objective values	51	4.5E13	5.81E13	7.11E13	t(50) = -12.28 p < .001	-1.72	-3.75	-2.56	D = .16, p < .001
Subjective values	51	3.9E8	4.73E8	5.25E8	t(50) = -8.60, p < .001	-1.2	-2.8	-1.62	D = .08, p = 1
Study 1 full									
Objective values	50	326.18	320.01	284.2	t(49) = -4.35, p < .001	- .61	-1.84	-0.56	D = .09, p = .72
Subjective values	50	.48	.37	.27	t(49) = 1.37, p = .36	.19	-0.33	1.27	D = .07, p = 1
Study 2									
Objective values	151	149.5	122.97	95.03	t(150) = -3.93, p < .001	- .32	-0.32	-0.99	D = .04, p = 1
Subjective values	151	.15	.11	.08	t(150) = .62, p = 1	.05	0.05	-0.26	D = .06, p = .51
Study 3 10 options									
Objective values	75	1.81	1.43	1.08	t(74) = 2.28, p = .05	.26	0	0.98	D = .08, p = .42
Subjective values	75	6.99	5.78	4.49	t(74) = 2.89, p = .01	.33	0.16	1.36	D = .06, p = 1
Study 3 14 options									
Objective values	65	5056.4	5042.47	4529.57	t(64) = -5.10, p < .001	- .63	-2.3	-0.87	D = .05, p = 1
Subjective values	65	38.66	34.25	27.99	t(64) = -3.55, p < .001	- .44	-1.57	-0.34	D = .08, p = .78

Note: All p and confidence interval alpha values are Bonferroni corrected for numbers of tests per study. *r* values columns represent the Cauchy scale factor of the prior on the effect size.

Supplementary Table 2

Details of tests between pairs of mean BIC values in pilot studies

Model 1	Model 2	*n*	Bayes factor 10 (BF10)			Cohen's *d*
			r = .5	*r* = .71	*r* = 1	
Pilot baseline						
Biased Prior obj	Cut Off obj	47	3.49E6	4.10E4	4.37E6	-1.66
Biased Prior obj	Cost to Sample obj	47	0.26	0.19	0.14	- .32
Cut Off obj	Cost to Sample obj	47	4.91E8	6.07E8	6.88E8	1.42
Pilot full						
Biased Prior obj	Biased Prior subj	50	0.21	0.16	0.11	0.02
Biased Prior obj	Cut Off obj	50	0.48	0.36	0.27	-0.19
Biased Prior obj	Cut Off subj	50	1.86	1.52	1.17	-0.32
Biased Prior obj	Cost to Sample obj	50	2.4	1.99	1.55	-0.34
Biased Prior obj	Cost to Sample subj	50	0.22	0.16	0.11	0.03
Biased Prior subj	Cut Off obj	50	0.34	0.25	0.19	-0.15
Biased Prior subj	Cut Off subj	50	0.5	0.38	0.29	-0.2
Biased Prior subj	Cost to Sample obj	50	0.24	0.18	0.13	-0.08
Biased Prior subj	Cost to Sample subj	50	0.21	0.15	0.11	-0.01
Cut Off obj	Cut Off subj	50	0.52	0.4	0.29	-0.2
Cut Off obj	Cost to Sample obj	50	0.24	0.18	0.13	0.08
Cut Off obj	Cost to Sample subj	50	0.51	0.39	0.29	0.2
Cut Off subj	Cost to Sample obj	50	0.57	0.44	0.33	0.21
Cut Off subj	Cost to Sample subj	50	2.69	2.23	1.75	0.34
Cost to Sample obj	Cost to Sample subj	50	0.93	0.73	0.55	0.26

Note. All *p* values Bonferroni corrected for the number of tested pairs per study. *r* values in the Bayes Factor columns represent the scale factor of the Cauchy prior on the effect size. Compare to middle row of Figure 3. Abbreviations: Subj = Models that make choices about subjective values; Obj = Models that make choices about objective values.

Supplementary Table 3.
Lilliefors tests of deviations from normality of sampling rates in Study 1 conditions

Condition	n	D	p
Baseline	50	.09	1
Squares	50	.08	1
Timing	50	.12	.42
Payoff	51	.1	1
Ratings	51	.11	.72
Full	50	.09	1

Note. All *p* values are Bonferroni corrected for tests of six conditions.

Supplementary Figure 1. Pilot full and Study 1 full paradigms

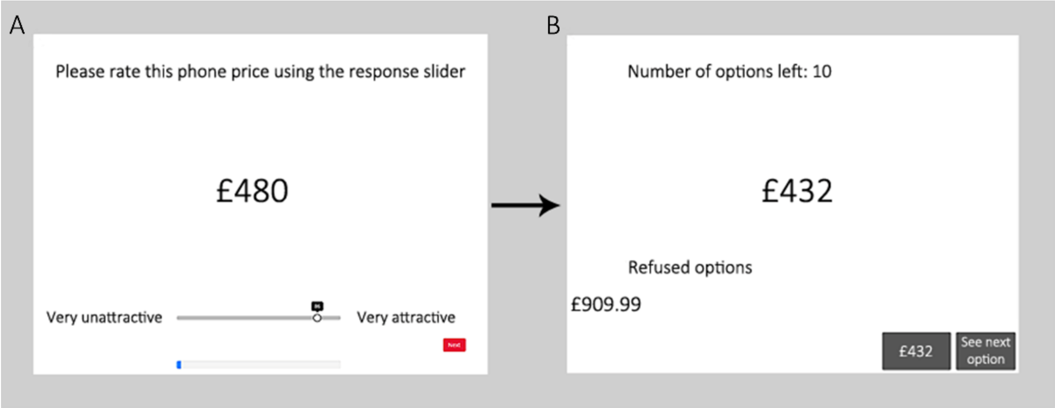

In an initial phase participants rate potential options for their subjective value (A). Then, in (B), participants view option screens as part of the optimal stopping decision task.

Supplementary Figure 2. Study 1 payoff condition

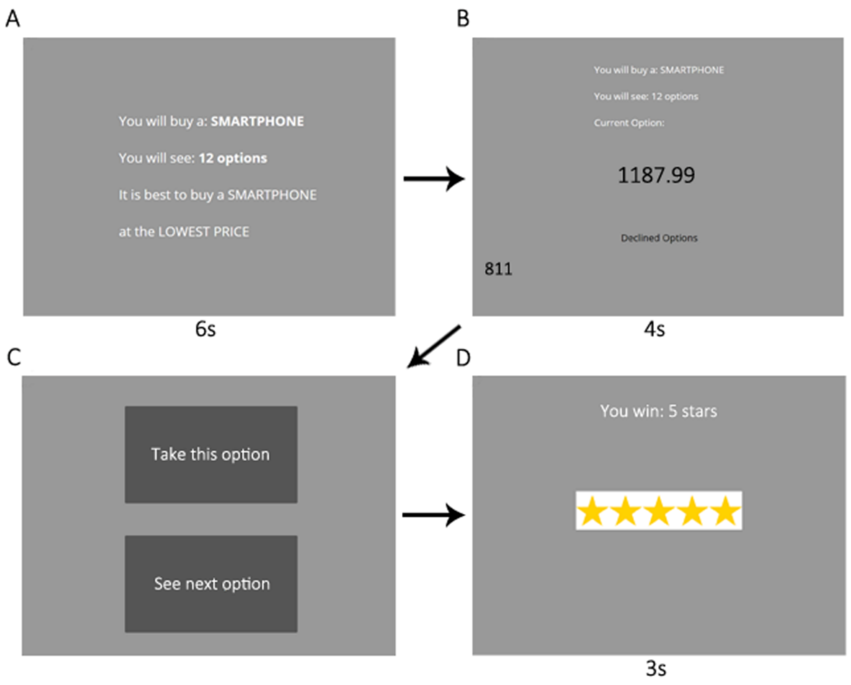

Participants receive instructions (A) before each new sequence of options screens (B), for which they choose to take each option or sample another (C). In the payoff condition, taking an option leads to feedback about the reward value of the choice, in the form of a number of stars that have been won.

Supplementary Figure 3. Study 1 squares condition

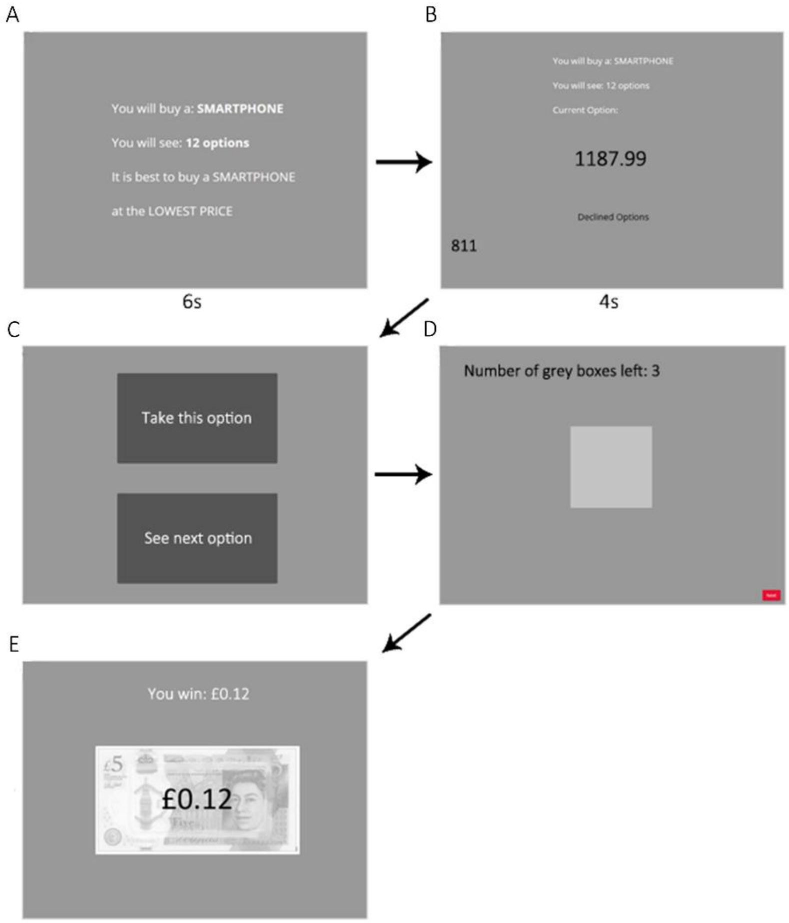

After receiving instructions (A), participants view option screens (B), followed by choices (C) to take the option or sample another. In the squares condition, when a choice is made, participants then must page through grey squares (D), which replace the remaining option screens, before they can continue to receive feedback on their choice in that sequence (E).

Supplementary Figure 4. Recovered parameters

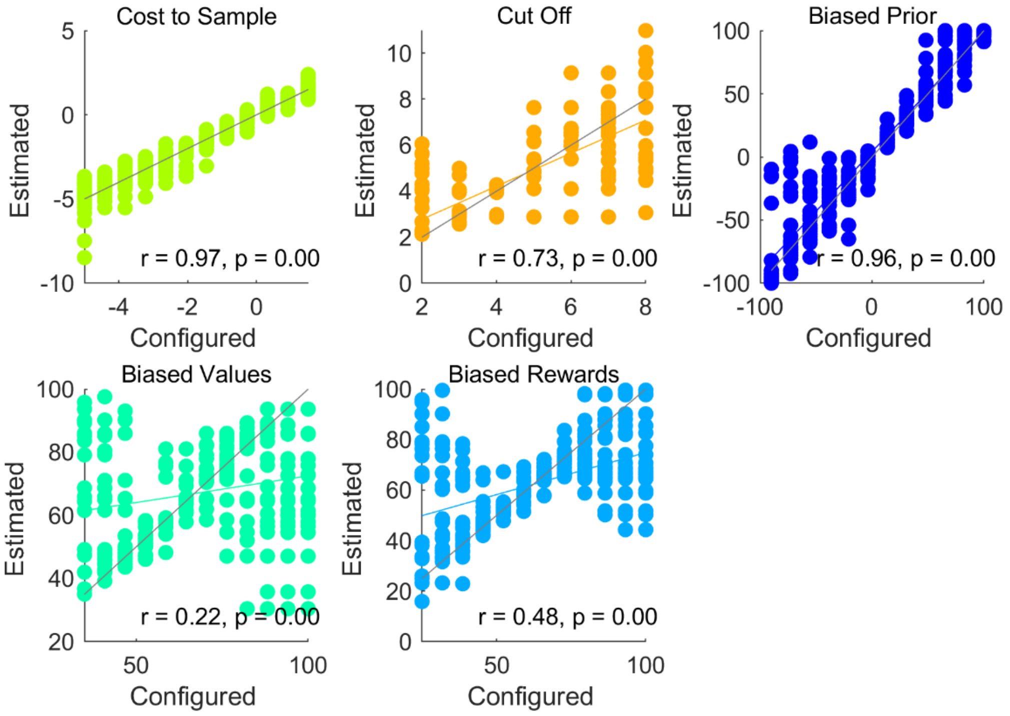

For parameter recovery analysis, we examined parameter values (vertical axes) estimated from fitting models to decisions simulated using configured parameter values (horizontal axes) for simulated participants, each shown as an individual scatter point. The grey diagonal indicates when configured and estimated parameters would be exactly equal. The coloured line indicates the regression line relating configured and estimated parameter values. Each regression is based on n = 300 observations (12 configured parameter levels × 25 simulated participants). Biased Rewards and Biased Values models showed too poor parameter recovery to be entered into formal model comparison.

Supplementary Figure 5. Parameter recovery details

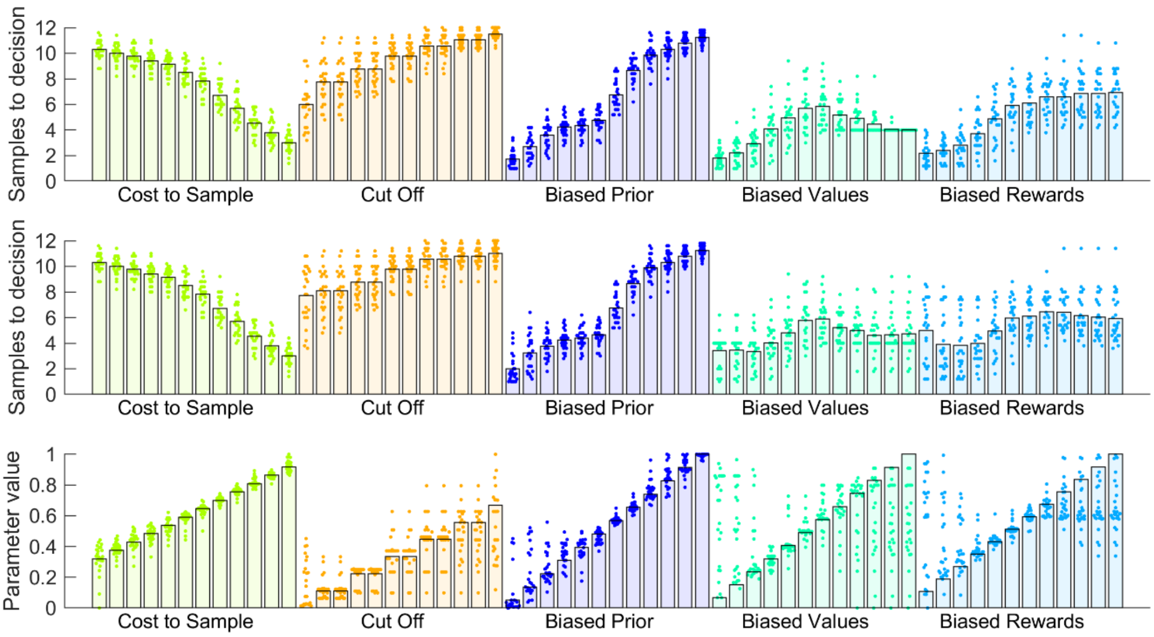

Top panel: We plot sampling rates for the 25 individual simulated participants (points) and their mean values (bars) for each of the 12 configured parameter level in the parameter recovery analysis. Systematically varying configured parameter values successfully increases or decreases simulated sampling rates for Cost to Sample and Biased Prior models and the Cut Off heuristic. Middle panel: Models were fitted to the data in the top panel and parameters estimated. We plot the sampling rates simulated using each estimated parameter (points) and their mean sampling rates (bars). Lower panel: The estimated parameters (points) are plotted relative to their target configured parameter values (bars). Each model's parameter values are normalised to a 0 to 1 range to facilitate plotting on one scale.

Supplementary Figure 6. Parameter recovery sampling rates

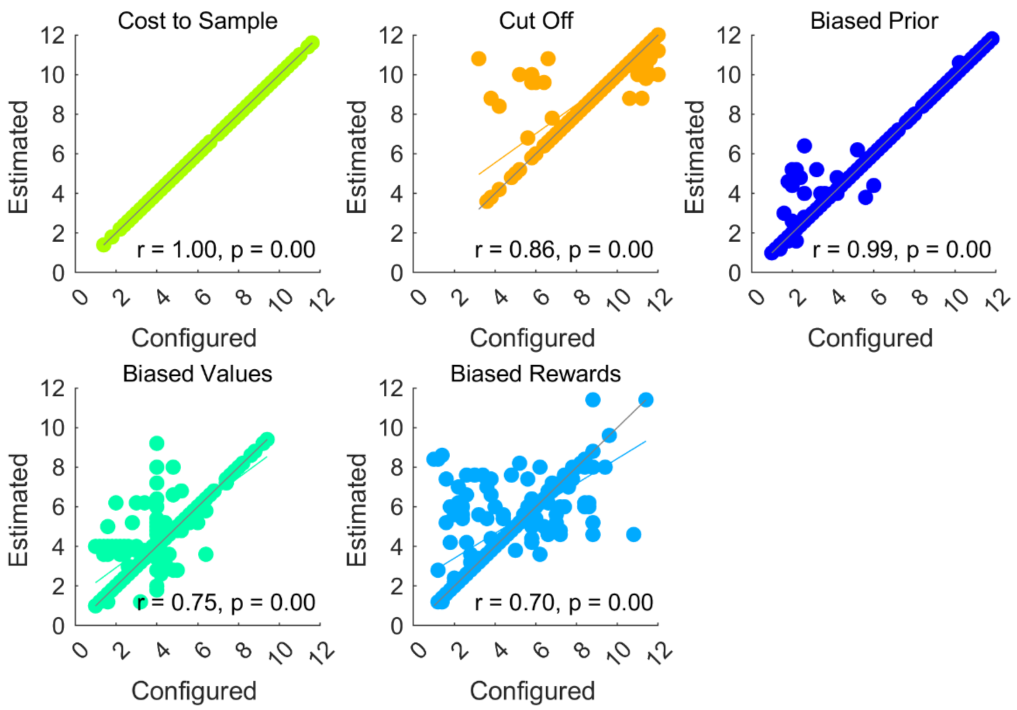

Sampling rates simulated using configured parameters (horizontal axis) are plotted against sampling rates computed from estimated parameters. The grey diagonal indicates when sampling rates based on configured and estimated parameters would be exactly equal. The coloured line indicates the regression line relating sampling rates based on configured and estimated parameter values. Each regression is based on *n* = 300 observations (12 configured parameter levels × 25 simulated participants).

Supplementary Figure 7. Model recovery

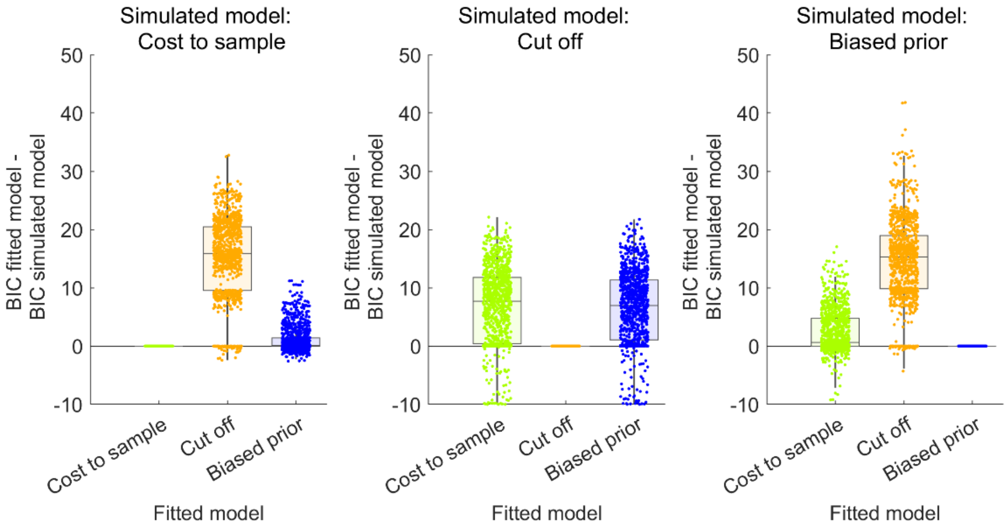

Choice data were simulated using the three models. Shown are Bayesian information criterion (BIC) values for each fitted model with the BIC value for the fitted model corresponding to the simulated model subtracted. The results show fits were largely better when the fitted and the simulated model were the same, suggesting successful model recovery.

Supplementary Figure 8. Supplementary analysis of pilot studies

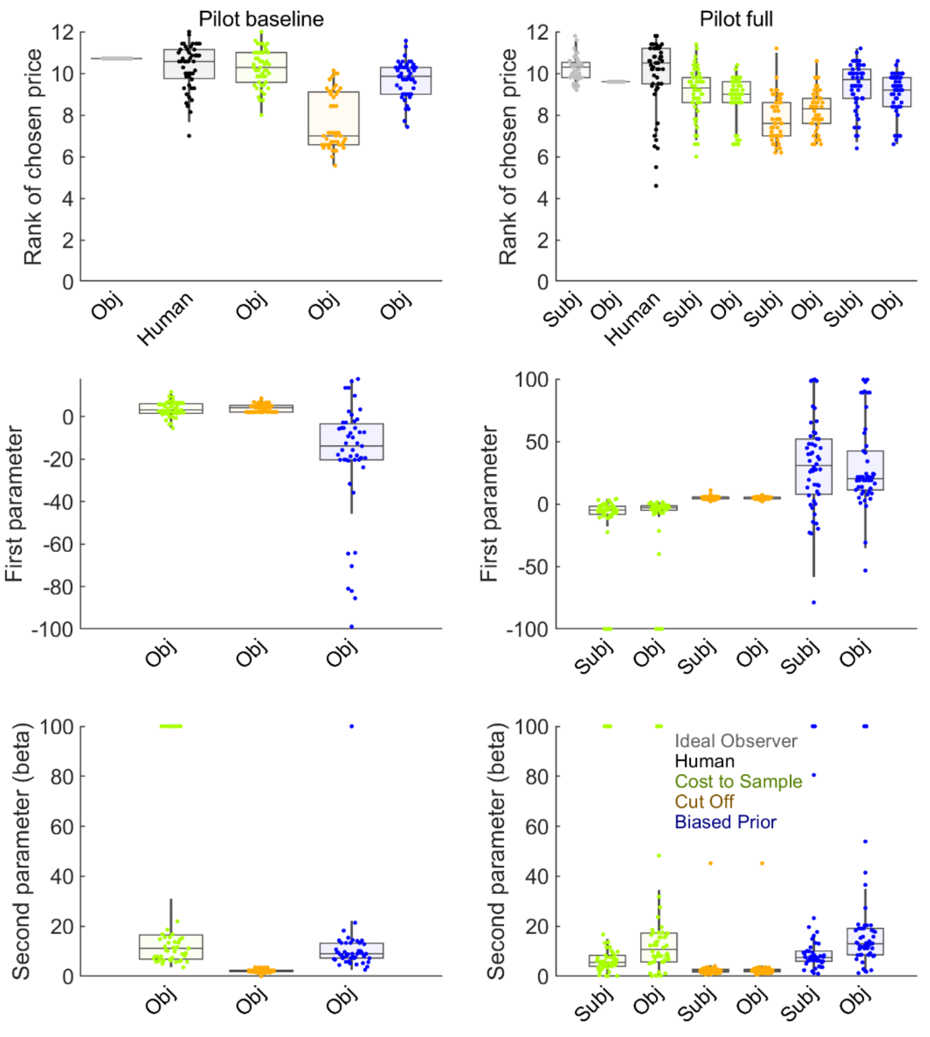

Model comparison for Pilot baseline (*n* = 47 participants) is in the left column and Pilot full (*n* = 50) is in the right column. The top row shows the ranks of chosen prices. The Cut Off model was not able to approximate participants' achieved ranks. The second row plots the "first" or key theoretical parameter values, estimated for each fitted model. For pilot baseline, where there was clear undersampling (Figure 3), the Biased Prior parameters tend negatively, suggesting a pessimistic outlook. The third row shows the "second," or inverse temperature parameter beta, estimated for each fitted model. Boxplots reflect first, second (median) and third quartiles, while whiskers reflect 1.5 interquartile range. Points reflect individual participant mean values. Abbreviations: Subj = Models that make choices about subjective values; Obj = Models that makes choices about objective values.

Supplementary Figure 9. Supplementary analysis of Study 1 conditions with no first phase

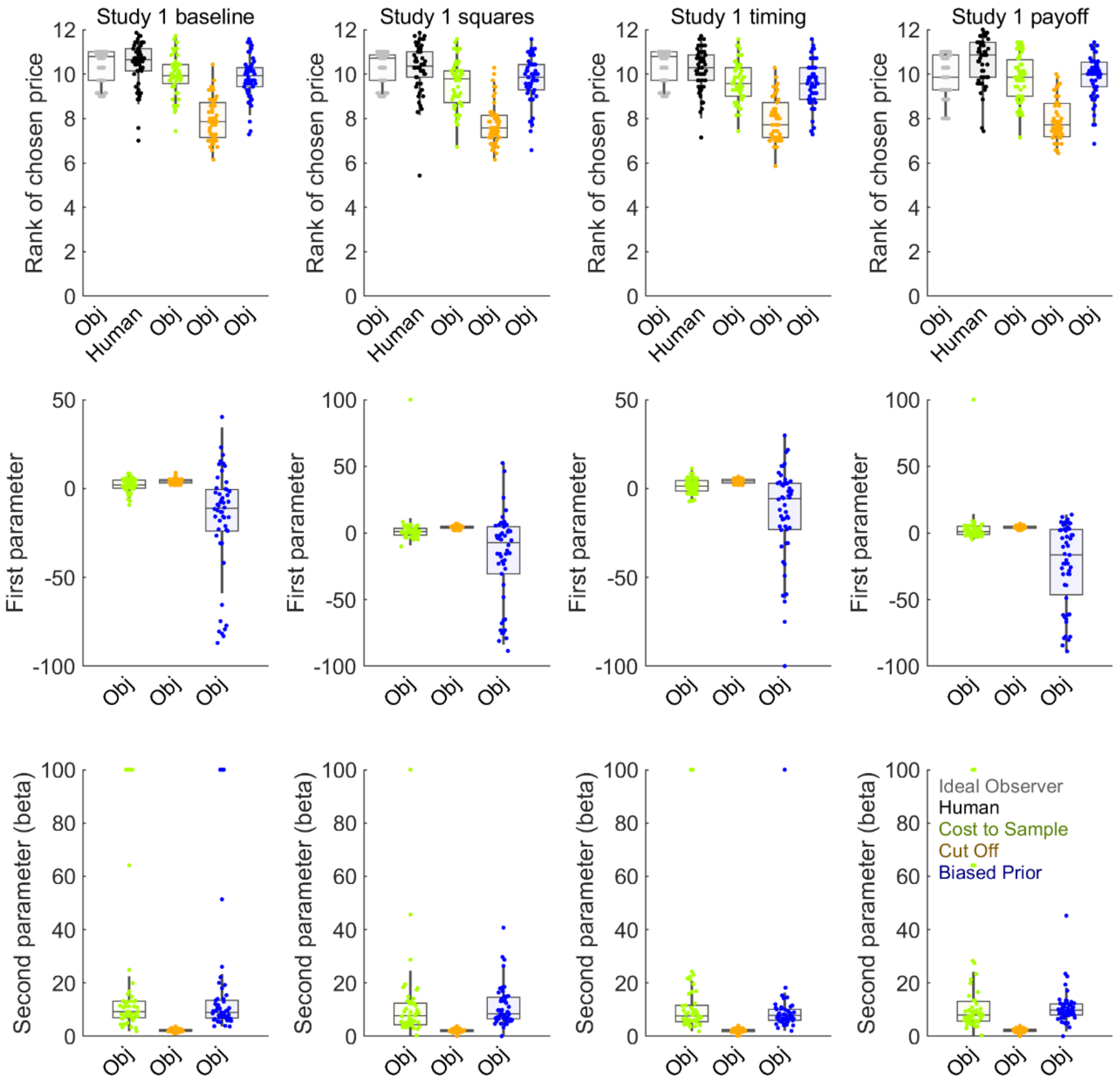

From left to right, columns show results from baseline (*n* = 50 participants), squares (*n* = 50), timing (*n* = 50) and payoff (*n* = 51) conditions. The top row shows ranks of chosen prices. The Cut Off model could never approximate participants' achieved ranks in any condition. The second row plots the "first" or theoretical parameter values, estimated for each fitted model. Every condition's Biased Prior model shows a negative (pessimistic) parameter, likely associated with undersampling in these conditions (Figure 4). The third row shows the "second," or inverse temperature parameter beta, estimated for each fitted model. Boxplots reflect first, second (median) and third quartiles, while whiskers reflect 1.5 interquartile range. Points reflect individual participant data. Abbreviations: Subj = Models that make choices about subjective values; Obj = Models that makes choices about objective values.

Supplementary Figure 10. Supplementary analysis of Study 1 conditions with a first phase

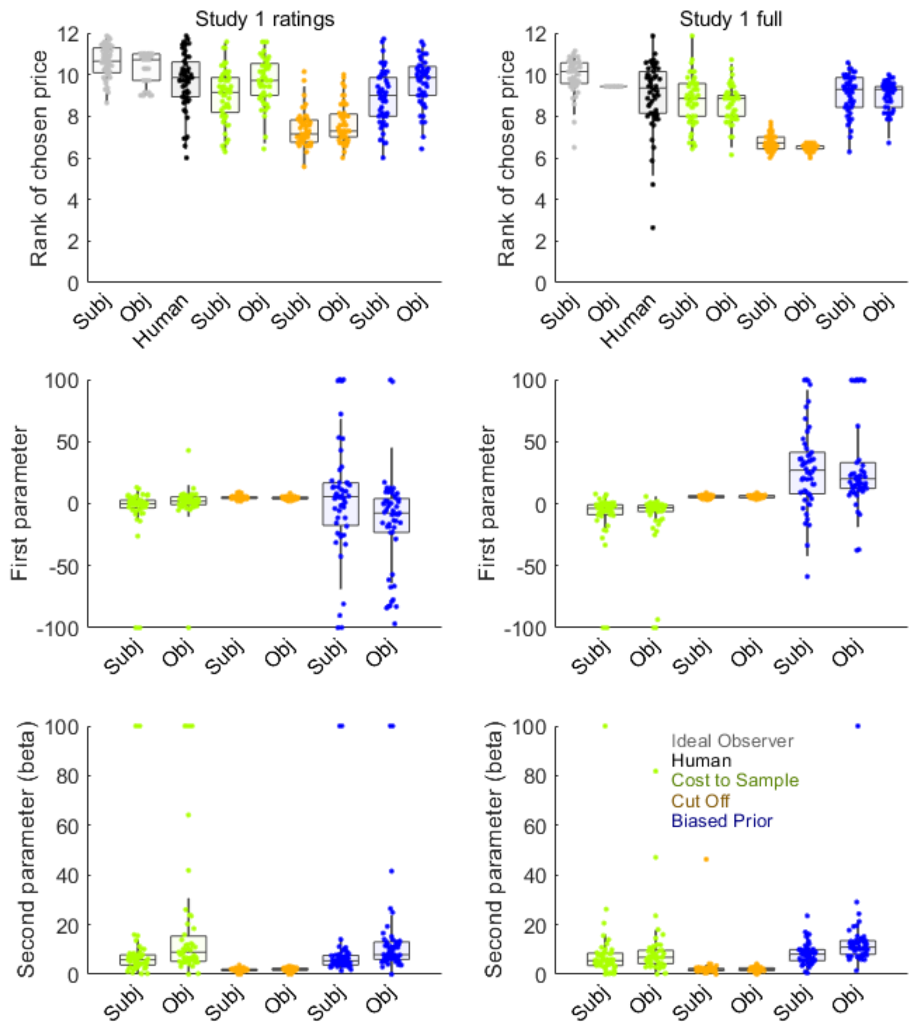

Results from the ratings condition (n = 51 participants) are shown in the left column and from the full condition (n = 50) on the right. The top row shows the ranks of chosen prices. The Cut Off model was not able to approximate participants' achieved ranks. The second row plots the "first" or theoretical parameter values, estimated for each fitted model. The condition showing the clearest undersampling, ratings (Figure 5) shows more negative (pessimistic) Biased prior parameter values. The third row shows the "second," or inverse temperature parameter beta, estimated for each fitted model. Boxplots reflect first, second (median) and third quartiles, while whiskers reflect 1.5 interquartile range. Points reflect individual participant data. Abbreviations: Subj = Models that make choices about subjective values; Obj = Models that makes choices about objective values.

Supplementary Figure 11. Supplementary analysis of Study 2

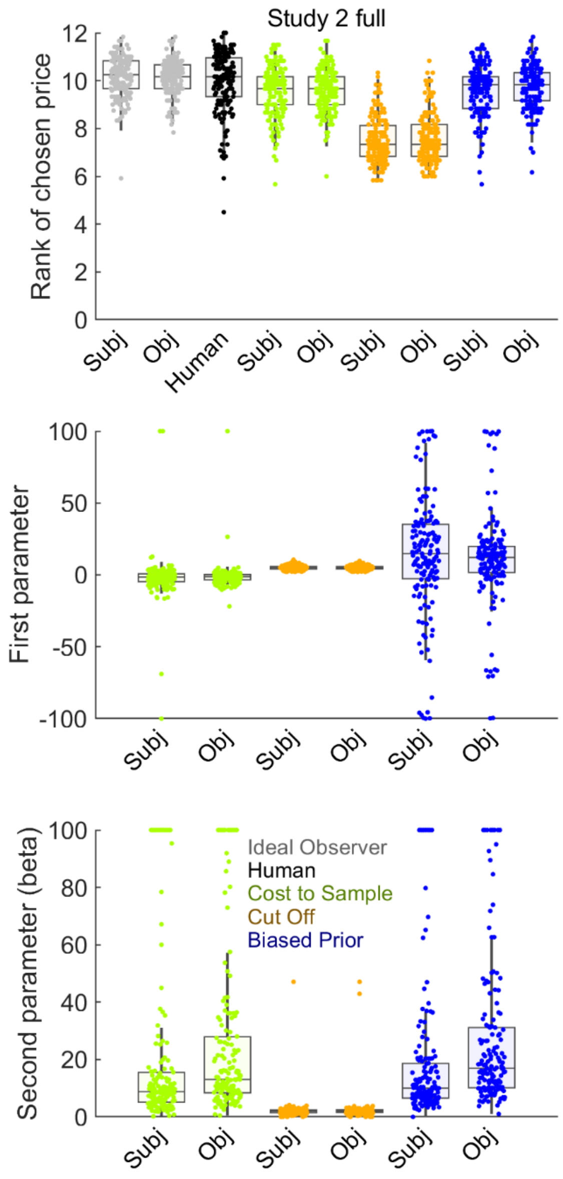

The top row shows ranks of chosen prices for *n* = 151 participants. The top row shows the ranks of chosen prices. The Cut Off model was not able to approximate participants' achieved ranks. The second row plots the "first" or theoretical parameter values, estimated for each fitted model. The third row shows the "second," or inverse temperature parameter beta, estimated for each fitted model. Boxplots reflect first, second (median) and third quartiles, while whiskers reflect 1.5 interquartile range. Points reflect individual participant data. Abbreviations: Subj = Models that make choices about subjective values; Obj = Models that makes choices about objective values.

Supplementary Figure 12. Model predictions of sampling rates in Study 2

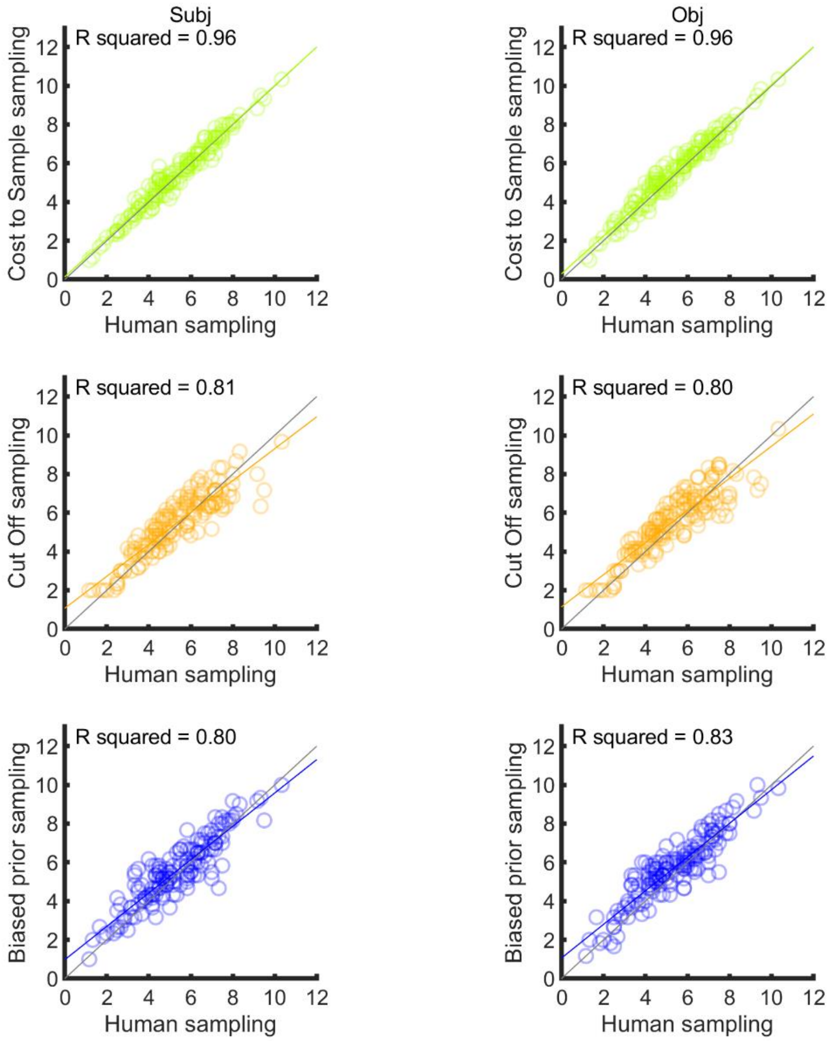

Relationships between individual differences in fitted model sampling and participant sampling for models (rows) operating on subjective values (left column) or objective values (right column). All models can predict human sampling from the *n* = 151 participants reasonably well. Abbreviations: Subj = Models that make choices about subjective values; Obj = Models that makes choices about objective values.

Supplementary Figure 13. Choice thresholds in Study 2

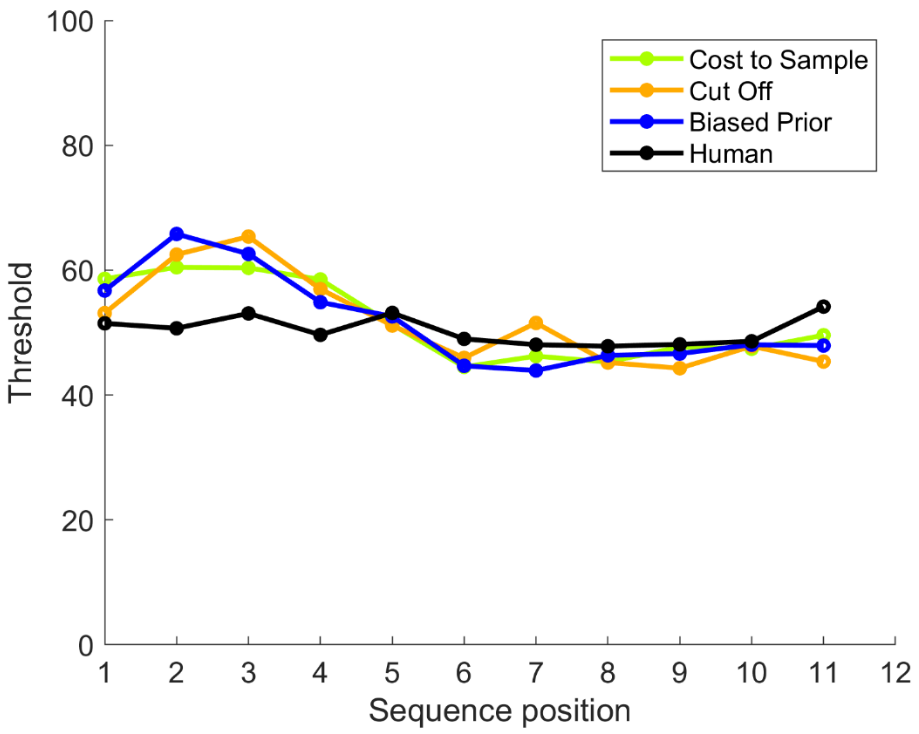

Decision thresholds (option values above which search is discontinued and the current option chosen) estimated for *n* = 151 participants and corresponding fitted models and averaged for each sequence position. Outside of the first few sequence positions, all models reasonably approximate participants' linearly-changing thresholds. In this example, models processed objective values of prices. Thresholds are estimated by fitting a model resembling the independent threshold model[3], which estimates each sequence position's threshold as a free parameter. Fitted data were either participants' choices or choices simulated by our models. Fitting was performed using fminearch in MATLAB. Though we recognise that choice thresholds are already explicitly computed within all our models and could be directly extracted from them, this threshold estimation procedure facilitated direct comparison with participants, by using the same procedure to estimate thresholds for participants and for models.

Supplementary Figure 14. Supplementary analysis of Study 3

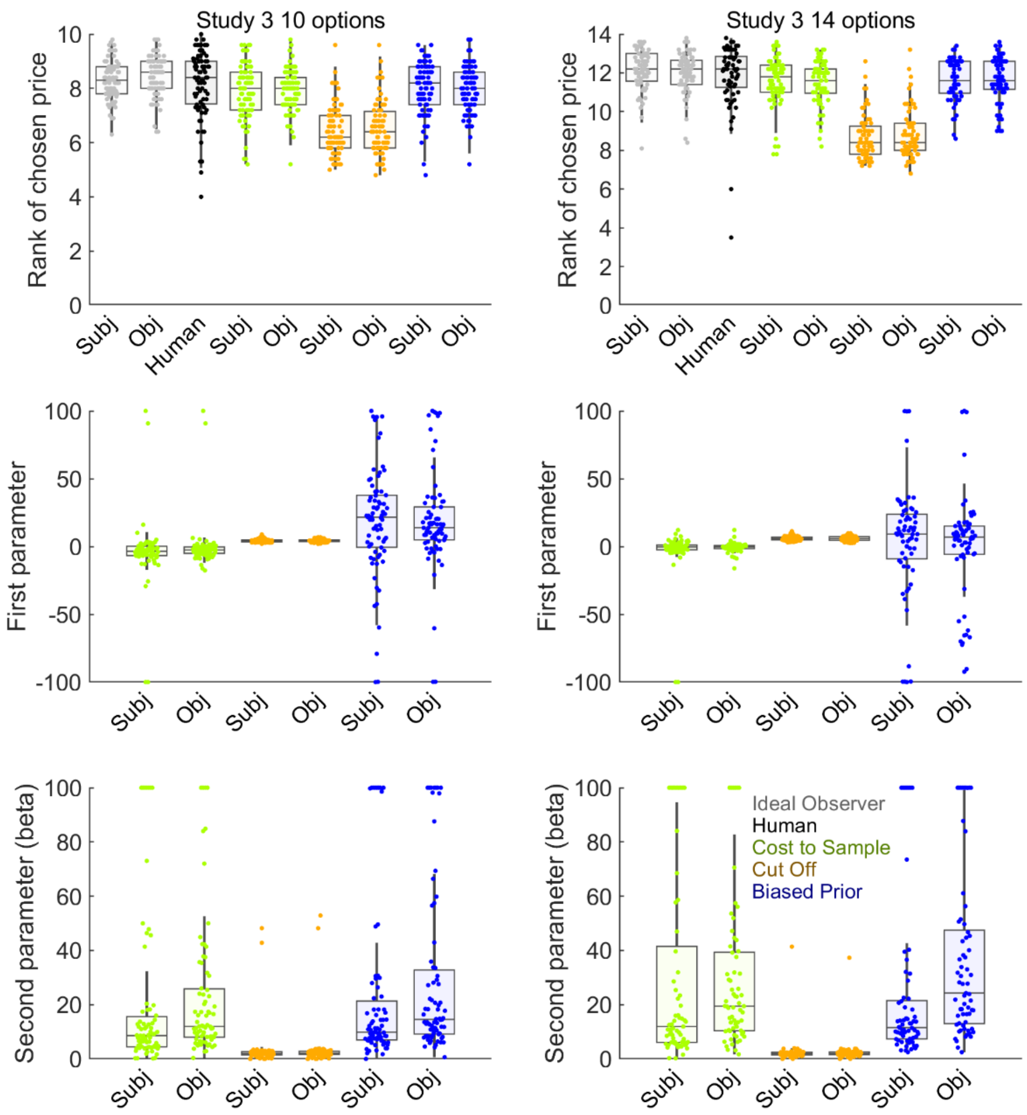

The left columns shows the 10 option condition (n = 75 participants) and the right column shows the 14 option condition (n = 65). The top row shows ranks of chosen prices, where the Cut Off model underperforms, compared to participants. The second row plots the "first" or theoretical parameter values, estimated for each fitted model. The third row shows the "second," or inverse temperature parameter beta, estimated for each fitted model. Boxplots reflect first, second (median) and third quartiles, while whiskers reflect 1.5 interquartile range. Points reflect individual participant data. Abbreviations: Subj = Models that make choices about subjective values; Obj = Models that makes choices about objective values.
