# Supplementary material for: Biased expectations about future choice options predict sequential economic decisions
Source: Commun Psychol. 2024 Dec 18;2:119. doi: 10.1038/s44271-024-00172-8 (PMC11655870; doi:10.1038/s44271-024-00172-8)
Supplement: Supplementary file 3 — Reporting Summary [file 44271_2024_172_MOESM3_ESM.pdf]

nature portfolio

Corresponding author(s): Nicholas Furl

Last updated by author(s): Jul 18, 2024

Reporting Summary

Nature Portfolio wishes to improve the reproducibility of the work that we publish. This form provides structure for consistency and transparency in reporting. For further information on Nature Portfolio policies, see our Editorial Policies and the Editorial Policy Checklist.

Statistics

For all statistical analyses, confirm that the following items are present in the figure legend, table legend, main text, or Methods section.

n/a	Confirmed	
☐	☒	The exact sample size (*n*) for each experimental group/condition, given as a discrete number and unit of measurement
☐	☒	A statement on whether measurements were taken from distinct samples or whether the same sample was measured repeatedly
☐	☒	The statistical test(s) used AND whether they are one- or two-sided<br>*Only common tests should be described solely by name; describe more complex techniques in the Methods section.*
☐	☒	A description of all covariates tested
☐	☒	A description of any assumptions or corrections, such as tests of normality and adjustment for multiple comparisons
☐	☒	A full description of the statistical parameters including central tendency (e.g. means) or other basic estimates (e.g. regression coefficient) AND variation (e.g. standard deviation) or associated estimates of uncertainty (e.g. confidence intervals)
☐	☒	For null hypothesis testing, the test statistic (e.g. *F*, *t*, *r*) with confidence intervals, effect sizes, degrees of freedom and *P* value noted<br>*Give P values as exact values whenever suitable.*
☐	☒	For Bayesian analysis, information on the choice of priors and Markov chain Monte Carlo settings
☐	☒	For hierarchical and complex designs, identification of the appropriate level for tests and full reporting of outcomes
☐	☒	Estimates of effect sizes (e.g. Cohen's *d*, Pearson's *r*), indicating how they were calculated

Our web collection on statistics for biologists contains articles on many of the points above.

Software and code

Policy information about availability of computer code

Data collection	Gorilla Experimental Builder https://gorilla.sc/<br>jspsych7.31 https://github.com/nicholasfurl/Model_fitting_hybrid_study/tree/main/javascript_paradigm
Data analysis	https://github.com/nicholasfurl/Model_fitting_hybrid_study/tree/main

For manuscripts utilizing custom algorithms or software that are central to the research but not yet described in published literature, software must be made available to editors and reviewers. We strongly encourage code deposition in a community repository (e.g. GitHub). See the Nature Portfolio guidelines for submitting code & software for further information.

Data

Policy information about availability of data

All manuscripts must include a data availability statement. This statement should provide the following information, where applicable:

- Accession codes, unique identifiers, or web links for publicly available datasets
- A description of any restrictions on data availability
- For clinical datasets or third party data, please ensure that the statement adheres to our policy

Data and code availability: https://github.com/nicholasfurl/Model_fitting_hybrid_study

Research involving human participants, their data, or biological material

Policy information about studies with human participants or human data. See also policy information about sex, gender (identity/presentation), and sexual orientation and race, ethnicity and racism.

Reporting on sex and gender	We do not have sex or gender information available for our participant samples and we did not ask for self-reports of sex or gender. We recruited participants from an online participant recruitment platform. We did not screen for sex or gender but did screen for UK participants only, and expect our sample should reflect the sex and gender distribution of that platform in the UK.
Reporting on race, ethnicity, or other socially relevant groupings	We did not categorise participants into social groupings, race or ethnicity.
Population characteristics	See below.
Recruitment	Participants were subscribed members of the commonly-used commercial online research participant recruitment platform Prolific. In addition to being subscribers, participants would need to select and volunteer for the study.
Ethics oversight	Royal Holloway, University of London College Ethics System

Note that full information on the approval of the study protocol must also be provided in the manuscript.

Field-specific reporting

Please select the one below that is the best fit for your research. If you are not sure, read the appropriate sections before making your selection.

☐ Life sciences ☒ Behavioural & social sciences ☐ Ecological, evolutionary & environmental sciences

For a reference copy of the document with all sections, see nature.com/documents/nr-reporting-summary-flat.pdf

Behavioural & social sciences study design

All studies must disclose on these points even when the disclosure is negative.

Study description	Quantitative
Research sample	*State the research sample (e.g. Harvard university undergraduates, villagers in rural India) and provide relevant demographic information (e.g. age, sex) and indicate whether the sample is representative. Provide a rationale for the study sample chosen. For studies involving existing datasets, please describe the dataset and source.*
Sampling strategy	All studies were random samples of volunteers from Prolific, a commonly-used participant recruitment platform. We screened for participants from the United Kingdom, as our study involved understanding of prices denominated in GBP. Our samples reflected the age and gender distributions of participants from the United Kingdom available from the Prolific platform.
Data collection	The study was conducted online and participants could use the device and setting of their choice.
Timing	Pilot baseline 7 Oct 2019<br>Pilot full 10 Dec 2019<br>Study 1 22-24th Feb 2021<br>Study 2 29-31 March 2023<br>Study 3 4th April 2023
Data exclusions	No data were excluded from the analysis.
Non-participation	We do not have data on what participants viewed but did not continue past consent form.
Randomization	Participants were randomly allocated into study conditions.

Reporting for specific materials, systems and methods

We require information from authors about some types of materials, experimental systems and methods used in many studies. Here, indicate whether each material, system or method listed is relevant to your study. If you are not sure if a list item applies to your research, read the appropriate section before selecting a response.

Materials & experimental systems

n/a	Involved in the study
☒	☐ Antibodies
☒	☐ Eukaryotic cell lines
☒	☐ Palaeontology and archaeology
☒	☐ Animals and other organisms
☒	☐ Clinical data
☒	☐ Dual use research of concern
☒	☐ Plants

Methods

n/a	Involved in the study
☒	☐ ChIP-seq
☒	☐ Flow cytometry
☒	☐ MRI-based neuroimaging

Plants

Seed stocks	N/A
Novel plant genotypes	N/A
Authentication	N/A
